# Readmission of late preterm and term neonates in the neonatal period

**DOI:** 10.1016/j.clinsp.2022.100005

**Published:** 2022-02-12

**Authors:** Darjan Kardum, Ivana Serdarušić, Borna Biljan, Krešimir Šantić, Vinko Živković

**Affiliations:** a Department of Pediatrics, University Hospital Osijek, Osijek, Croatia; b School of Medicine, University J. J. Strossmayer Osijek, Osijek, Croatia

**Keywords:** Newborn, Patient readmission, Length of stay, Patient discharge

## Abstract

**Objective:**

To determine the incidence of hospital readmissions in late preterm and term neonates, the most common reasons for readmission, and analyze the risk factors for readmission in the neonatal period.

**Methods:**

Newborn infants admitted to a well-baby nursery ≥ 36 weeks gestation were included in this retrospective cohort study. Data for all infants born in a 3-year period and readmitted in the first 28 days of life were analyzed. Indication for readmission was one diagnosed during initial workup in the pediatric emergency room visit before readmission.

**Results:**

The final cohort consisted of 5408 infants. The readmission rate was 4.0% (219/5408). Leading readmission causes were respiratory tract infection (29.58%), jaundice (13.70%), and urinary tract infection (9.59%). The mean ± SD age of readmitted infants was 13.3 ± 7.1 days. The mean ± SD treatment duration of treatment was 5.5 ± 3.0 days. In the multivariate regression analysis, infants that were during the initial hospitalization transferred to special care/NICU had a lower chance of readmission during the neonatal period (p = 0.04, OR = 0.23, 95% CI 0.06–0.93). Infants with mothers aged from 19–24 years had a higher risk of readmission (p = 0.005, OR = 1.62, 95% CI 1.16–2.26).

**Conclusions:**

Finding that infants that were during the initial hospitalization transferred to special care or a NICU setting were less likely to require hospitalization in the neonatal period is an interesting one. Further research into how different approach in these settings reduce the risk of readmission is necessary.

## Highlights

• In infants gestational age ≥ 36 weeks the readmission rate is 4.0%.
• Most common causes are respiratory infections, jaundice, and feeding problems.
• Initial care in special care or NICU setting showed reduced readmission rates.

## Introduction

Readmission of neonates in the neonatal period has in the past decades come into focus primarily due to its connection with growing trends of early discharge after birth (<48 hours in vaginally born infants). Since the 1970s, there have been increasing trends of shorter hospital stays for infants after uncomplicated vaginal birth and following C-section,^1^ mainly due to increasing healthcare costs.^2^

A meta-analysis of 10 trials found that earlier postnatal discharge of healthy mothers and term infants do not appear to have adverse effects on breastfeeding or maternal depression when accompanied by a policy of offering women at least one nurse-midwife home visit post-discharge. Trends favoring the early discharge group were observed for all maternal and infant outcomes except for maternal and infant readmissions, which favored the standard care group.^3^

Fahrat et al.^4^ reported that hospital discharge at any time ≤48 hours significantly increases the risk for readmission as well as the risk for readmission due to hyperbilirubinemia. A report from Saudi Arabia^5^ found that hospital discharge of neonates within 48 hours after delivery significantly increases the risk for hospital readmission during the neonatal period with the pre-dominance of sepsis-related cases.

In most cases, newborns are readmitted due to jaundice^6^ and infections.^5^^,^^6^ Several factors have been identified to contribute to readmissions: exclusively breastfed neonates,^1^ community nurse visit after > 72 hours,^7^ ethnic background,^8^ maternal age, and a number of previous deliveries.^9^

In Croatia, there are no official recommendations for the discharge of the mother-infant dyad, but vaginally born infants are in general discharged after 48–72 hours and 5–7 days following birth by cesarean section. After discharge, every neonate is visited by a community nurse on the first working day of the week. In the studied county, primary, secondary and tertiary care for children <18 years of life is readily accessible, and all treatment for children is fully covered by national health insurance.

In this study, the authors aimed to determine the incidence of hospital readmissions in late preterm and term neonates, the most common reasons for readmission, and analyze the risk factors for readmission in the neonatal period.

## Materials and methods

This retrospective cohort study was performed at a regional university hospital with a level 3 neonatal unit, special care unit, and well-baby nursery. The authors reviewed the medical charts of all live-born infants with gestational age (GA) at birth ≥ 36 weeks from January 2017 to December 2019 admitted to the well-baby nursery.

Data were analyzed for all infants readmitted after discharge from the initial hospitalization until 28 days of life that were admitted to a single university hospital. Among the data gathered for the newborns were age, sex, birth weight, length of initial hospitalization, treatment during initial hospitalization, diagnosis on admission, duration of treatment, the timing of first community nurse visit. Data for their mothers were also obtained. Among these were the age of the mother, type of delivery, number of previous deliveries, education level, and place of residence. Indication for readmission was one diagnosed during initial workup in the pediatric emergency room visit before readmission.

Ethics: The study was approved by the University Hospital Osijek Ethics committee (n° R2-13170/2020).

### Statistical analysis

Data were described by using descriptive statistical methods. Differences in categorical variables were tested by the Chi-Square test. The normality of the distribution of numerical variables was tested by the Shapiro-Wilk test. Numerical data were described by the median and the limits of the Interquartile Range (IQR). Differences between numerical variables between two independent groups were tested by the Mann-Whitney *U* test. Logistic regression (univariate and multivariate) was used to analyze the independent factors associated with the factors influencing readmission. The level of significance was set at 0.05. Statistical analysis was performed using SPSS 17.0 (SPSS Inc., Chicago, IL, USA), and MedCalc® Statistical Software version 20 (MedCalc Software Ltd, Ostend, Belgium; https://www.medcalc.org; 2021).

## Results

The initial cohort included 5454 infants with gestational age ≥36 weeks gestation, and 46 infants were immediately after birth admitted to the neonatal intensive care unit and were not included in the analysis. The final cohort consisted of 5408 infants. The overall readmission rate was 4.0% (219/5408) for infants ≤ 28 days of life and admitted for non-elective reasons. The leading cause for readmission was respiratory tract infection (29.68%), jaundice (13.70%), feeding difficulties or vomiting (10.96%), urinary tract infection (9.13%), and umbilical cord issues (6.85%). Other reasons for readmission are listed in Table 1.

**Table 1:** Reasons for readmission.

Condition	n	%
Respiratory tract infection	65	29.68%
Jaundice	30	13.70%
Feeding difficulties, vomiting	24	10.96%
Urinary tract infection	20	9.13%
Umbilical cord issues	15	6.85%
Skin conditions	11	5.02%
Suspected seizures	11	5.02%
Poor weight gain	11	5.02%
Parental concern	10	4.57%
Enterocolitis	7	3.20%
Fever without localizing sign	7	3.20%
Localized infection	3	1.37%
Aspiration	3	1.37%
Blood disorder	1	0.46%
Trauma	1	0.46%
Total	219	100.00%

The mean ± SD age of readmitted infants was 13.3±7.1 days. The mean ± SD treatment duration of treatment was 5.5 ± 3.0 days. Comparisons of non – readmitted and readmitted infants and the differences between cohorts are shown in Table 2.

**Table 2:** Characteristics of all non – readmitted and readmitted infants.

Characteristic	Not readmitted infants (n = 5189)	Readmitted infants (n = 219)	All infants (n = 5408)	p-value^a^
Gestational age, completed weeks, [Median (min – max)]	39 (36 – 43)	39 (36 – 42)	39 (36 – 43)	0.06^b^
Birth weight, grams [Median (IQR)]	3410 (3090 – 3720)	3460 (3140 – 3720)	3410 (3090–3720)	0.41^b^
< 2499 g, [n (%)]	193 (3.7)	6 (2.7)	199 (3.7)	0.45
> 2500 g, [n (%)]	4996 (96.3)	213 (97.3)	5209 (96.3)	
Sex, female	2505 (48.3)	101 (46.1)	2606 (48.2)	0.53
Lenght of inital stay, days [Median (IQR)]	3 (3 – 5)	3 (3 – 4)	3 (3 – 5)	0.11^b^
≤ 4 days [n (%)]	3741 (72.1)	169 (77.2)	3910 (72.3)	0.10
≥ 5 days [n (%)]	1446 (27.9)	50 (22.8)	1496 (27.7)	
Community nurse visit, days [Median (IQR)]	1 (1 – 2)	1 (1 – 2)	1 (1 – 2)	0.47^b^
≤ 2 days [n (%)]	4181 (83.1)	185 (84.5)	4366 (83.2)	0.60
≥ 3 days [n (%)]	850 (16.9)	34 (15.5)	884 (16.8)	
5ʹ Apgar score [Median (min – max)]	10 (0 – 10)	10 (6 – 10)	10 (0 – 10)	0.96^b^
Born via C – section	1335 (25.7)	51 (23.3)	1386 (25.6)	0.42
During initial hospitalisation transfered to special care/NICU [n(%)]	190 (3.7)	2 (0.9)	192 (3.6)	0.02
Maternal age, years [Median (IQR)]	30 (27 – 34)	29 (25 – 33)	30 (27 – 34)	0.01^b^
≤ 18 [n (%)]	81 (1.6)	2 (0.9)	83 (1.5)	0.04
19 – 24 [n (%)]	740 (14.3)	46 (21)	786 (14.5)	
25 – 29 [n (%)]	1483 (28.6)	68 (31.1)	1551 (28.7)	
30 – 34 [n (%)]	1844 (36)	64 (29)	1908 (35)	
35 – 39 [n (%)]	872 (17)	30 (14)	902 (17)	
≥40 [n (%)]	169 (3)	9 (4)	178 (3)	
Primipara, yes [n (%)]	2359 (45.5)	110 (50.2)	2469 (45.7)	0.17
Number of previous deliveries [n (%)]				
0–1	4160 (80.2)	185 (84.5)	4345 (80.3)	0.14
2–3	883 (17)	32 (14.6)	915 (16.9)	
≥4	146 (2.8)	2 (0.9)	148 (2.7)	
Place of residence [n (%)]				
City	1911 (36.8)	79 (36.1)	1990 (36.8)	0.94
Other city	876 (16.9)	36 (16.4)	912 (16.9)	
Rural	2402 (46.3)	104 (47.5)	2506 (46.3)	
Exclusively breastfeed, yes [n (%)]	3883 (74.8)	165 (75.3)	4048 (74.9)	0.86
Complications during pregnancy, yes [n (%)]	1828 (35.2)	73 (33.3)	1901 (35.2)	0.57
Season of birth [n (%)]				
Spring	1191 (23)	37 (16.9)	1228 (22.7)	0.09
Summer	1449 (27.9)	66 (30.1)	1515 (28)	
Autumn	1311 (25.3)	52 (23.7)	1363 (25.2)	
Winter	1236 (24)	64 (29)	1300 (24)	
Maternal education [n (%)]				
Unknown	620 (12)	25 (11.4)	645 (11.9)	0.84
Elementary school	60 (1.2)	4 (1.8)	64 (1.2)	
High school	3119 (60.1)	131 (59.8)	3250 (60.1)	
Faculty	1387 (27)	59 (27)	1446 (27)	

a Chi-Squared test

b Mann Whitney *U* test

When comparing two groups of infants (Table 3), infants that were transferred to the NICU during the initial hospitalization have a lower chance of readmission compared to infants that were discharged home (p = 0.02, RR = 0.25, 95% CI = 0.06–0.99) and infants whose mothers are up to 29 years old have a higher chance of readmission (p = 0.01, RR = 0.85, 95% CI = 0.73–0.98).

**Table 3:** Comparison of non – readmitted and readmitted infants.

Characteristic	Not readmitted infants (n = 5189)	Readmitted infants (n = 219)	All infants (n = 5408)	p-value^a^	RR	95% CI of OR
Gestational age						
≤36 weeks	176 (3.4)	4 (1.8)	180 (3.3)	0.21	1.02	0.99 – 1.04
≥37 weeks	5013 (96.6)	215 (98.2)	5228 (96.7)			
Birth weight						
< 2499 g	193 (3.7)	6 (2.7)	199 (3.7)	0.45	1.01	0.99 – 1.03
> 2500 g	4996 (96.3)	213 (97.3)	5209 (96.3)			
Gender						
female	2505 (48.3)	101 (46.1)	2606 (48.2)	0.53	1.04	0.92 – 1.18
male	2684 (51.7)	118 (53.9)	2802 (51.8)			
Lenght of inital stay, days						
≤ 4 days	3741 (72.1)	169 (77.2)	3910 (72.3)	0.10	0.82	0.64 – 1.05
≥ 5 days	1446 (27.9)	50 (22.8)	1496 (27.7)			
Community nurse visit, days						
≤ 2 days	4181 (83.1)	185 (84.5)	4366 (83.2)	0.60	0.92	0.67 – 1.26
≥ 3 days	850 (16.9)	34 (15.5)	884 (16.8)			
Mode of delivery						
Vaginal birth	3854 (74.3)	168 (76.7)	4022 (74.4)	0.42	0.91	0.71 – 1.16
C - section	1335 (25.7)	51 (23.3)	1386 (25.6)			
Initial hospitalisation ended with:						
Home discharge	4999 (96.3)	217 (99.1)	5216 (96.4)	0.02	0.25	0.06 – 0.99
Transfer to special care/NICU	190 (3.7)	2 (0.9)	192 (3.6)			
Maternal age, years						
≤ 29	2304 (95.2)	116 (96.6)	2420 (44.7)	0.01	0.85	0.73 – 0.98
≥ 30	2885 (4.8)	103 (3.4)	2988 (55.3)			
Primipara						
No	2830 (54.5)	109 (49.8)	2939 (54.3)	0.17	1.10	0.97 – 1.26
Yes	2359 (45.5)	110 (50.2)	2469 (45.7)			
Exclusively breastfeed						
Yes	1306 (25.2)	54 (24.7)	1360 (25.1)	0.86	0.98	0.77 – 1.24
Other	3883 (74.8)	165 (75.3)	4048 (74.9)			
Complications during pregnancy						
No	3361 (64.8)	146 (66.7)	3507 (64.8)	0.57	0.95	0.78 – 1.15
Yes	1828 (35.2)	73 (33.3)	1901 (35.2)			

a Chi-squared test. RR, Relative Risk; 95% CI, Confidence Interval 95%.

In the univariate logistic regression analysis, no predictor was found to be significant for readmission (Table 4). The multivariate regression analysis (stepwise method) showed that infants that were during the initial hospitalization transferred to NICU had a lower chance of later readmission during the initial 28 days of life (p = 0.04, OR = 0.23, 95% CI = 0.06–0.93). Infants with mothers ages from 19–24 years had a higher risk of readmission in the first 28 days of life (p = 0.005, OR = 1.62, 95% CI = 1.16–2.26).

**Table 4:** Univariate logistic regression analysis of factors influencing readmission.

Predictors	β	St. error	Wald	p	OR	95% CI
Gestational age (≤ 36)	-0.64	0.51	1.55	0.21	0.53	0.19 to 1.44
Birth weight (< 2500)	-0.32	0.42	0.56	0.45	0.73	0.32 to 1.66
Male	0.09	0.14	0.39	0.53	1.09	0.83 to 1.43
Lenght of inital stay (≥ 5 days)	-0.27	0.16	2.66	0.10	0.77	0.56 to 1.06
Community nurse visit (≥ 3 days)	-0.10	0.19	0.28	0.60	0.90	0.62 to 1.31
C – section	-0.13	0.16	0.66	0.42	0.88	0.64 to 1.21
During initial hospitalisation transfered to special care/NICU	-1.42	0.71	3.94	0.05	0.24	0.06 to 0.98
Maternal age (< 18)						
19 – 24	0.92	0.73	1.59	0.21	2.52	0.60 to 10.6
25 – 29	0.62	0.73	0.73	0.39	1.86	0.45 to 7.71
30 – 34	0.34	0.73	0.22	0.64	1.41	0.34 to 5.84
35 – 39	0.33	0.74	0.20	0.65	1.39	0.33 to 5.94
≥ 40	0.77	0.79	0.94	0.33	2.16	0.46 to 10.2
Primipara (yes)	0.19	0.14	1.92	0.17	1.21	0.92 to 1.59
Exclusively breastfeed	-0.03	0.16	0.03	0.86	0.97	0.71 to 1.33
Maternal complications during pregnancy	-0.08	0.15	0.33	0.57	0.92	0.69 to 1.22

## Discussion

The history of opinions regarding the proper timing of discharge of healthy neonates is a complicated one. Length of postnatal hospital stay has declined dramatically in the past fifty years worldwide. In the United States in the 1970s, the average hospital stay after uncomplicated vaginal birth was 4.1 days and following delivery by cesarean section 7.8 days.^10^ In 1992 postnatal stays were reduced to 2.1 days following vaginal birth and 4 days after cesarean section.^10^ Worldwide, postnatal hospital stays vary significantly after singleton vaginal delivery, ranging from just 0.5 days in Egypt in the year 2008 to 6.2 days in Ukraine in 2007.^11^

Paper by Luciano emphasizes that too short a hospital stay can lead to higher readmission rates due to jaundice, feeding difficulties, hypernatraemic dehydration, infections, congenital heart defects, and gastrointestinal obstruction.^12^ Since the length of postnatal stay can significantly impact readmission rates and subsequently lead to increased neonatal mortality and morbidity^13^ rates of readmission, causes of readmission and factors influencing readmission should be understood in national, regional, and local contexts.

In the present study, the overall readmission rate for neonates ≥36 weeks gestation in the neonatal period is 4.0% (219/5454). This rate is slightly lower than in Slovenia in 2012 (6% in June 2012 and 5.9% in November 2012).^6^

Studies report readmission rates of 0.62% within the first ten days after discharge,^14^ 1.0% to 3.7% within two weeks after nursery discharge in infants of all gestation.^7^ A study by Metcalfe et al.^15^ found readmission rates of 4.6% in 2008–2010 among vaginal births and 2.4% among cesarean births in Canada (excluding Quebec).

An interesting outlier in these reports is a study by Kavehmanesh et al.^16^ with readmission rates of 12.6% just for hyperbilirubinemia in Iranian infants > 2500 g birth weight. The study reported a mean (SD) length of nursery stay was 30.2 (23.9) hours.

In the present study, the median (IQR) length of postnatal stay was 3 (3–5) days with no statistically significant difference in length of initial stay between readmitted and non-readmitted infants (p = 0.11).

Regarding the reason for readmission, the present study found interesting differences from earlier studies. In the present study, the five leading reasons for readmission were respiratory tract infection (29.68%), jaundice (13.70%), feeding difficulties or vomiting (10.96%), urinary tract infection (9.13%), and umbilical cord issues (6.85%). In contrast to the present study, the majority of reports classify jaundice as the leading cause of readmission.^6^^,^^14^^,^^16^ This may be due to the study's institutional rigorous clinical observance for hyperbilirubinemia during initial hospitalization.

In the present study, even though respiratory tract infections are the leading cause of readmission, the season of birth is not significantly different between readmitted and non- readmitted infants, excluding the causal connection with seasonal viral infections and timing of birth. Also, there are no differences between the two groups of infants regarding or not the infant has an older sibling (Table 2). Regarding this finding, the authors can only hypothesize that cultural context comes into view regarding well-established local customs of immediate family visits after discharge in Croatia, but the authors did not include this data in this study. Also, the local practice could influence the admission rates for respiratory tract infections since clinicians have a low threshold for hospitalization in the neonatal period.

Feeding difficulties are among the most common reasons for readmission reported earlier.^17^ In the study's cohort of readmitted infants, feeding difficulties and poor weight gain presented a significant proportion of readmitted infants. Although these conditions are sometimes associated with exclusively breastfed infants, and the readmission rate for breastfeeding problems is approximately 0.3% to 2% of term or near-term infants,^18^ the authors found no correlation between exclusivebreastfeeding rates in readmitted and non – readmitted neonates (Table 3), and exclusive breastfeeding was not found to be a risk factor for readmission (Table 4)

One specific of this study is that in the reasons examined, particular interest was placed on parental concerns as a reason for readmission. These concerns accounted for 4.57% of all readmissions. These infants were in all cases hospitalized for short workups and discharged mostly the next day. Even though these cases do not account for a large number of readmissions, they could indicate that a more methodical approach needs to be undertaken in preparation of the family for newborn discharge, similar to those proposed in the American Academy of Pediatrics recommendations from 2010.^19^ In accordance with the AAP recommendations,^20^ both cohorts of neonates were visited by a licensed health care professional. However, 83.2% were visited in the preferred 72 hours after discharge. This was not found to be a risk factor for readmission (Table 4)

When comparing both groups of infants, readmitted infants were more commonly born to mothers ≤ 29 years of age (p = 0.01, OR = 0.85, 95% CI = 0.73–0.98), and non – readmitted infants were more often hospitalized in special care or NICU setting (p = 0.02, OR = 0.25, 95% CI = 0.06–0.99). However, in the univariate analysis (Table 4), no single factor was identified to significantly contribute to readmission.

In the multivariate analysis, if the initial hospitalization continued with transfer to special care or NICU setting with subsequent discharge, these infants were less likely to be readmitted later on (p = 0.04, OR = 0.23, 95% CI = 0.06–0.93). This finding can be analyzed from several points of view. At first view, this finding is consistent with previous findings that a longer length of stay reduces the readmission rates.^20^ However, the duration of initial hospitalization did not significantly vary between readmitted and non-readmitted infants (Table 2).

In the study's institution, when an infant is transferred to special care or NICU setting, infants are hospitalized with their mothers, with a larger number of nursing staff and presumably more support regarding infant care. This all can contribute to alleviating common readmission issues and parental concerns (i.e., feeding difficulties, umbilical cord care) and could reduce the risk for readmission.

The only factor that was found to lead to a higher risk for readmission was maternal age from 19–24 years (p = 0.005, OR = 1.62, 95% CI = 1.16–2.26). Interestingly, maternal age ≤ 18 years was not found to be a significant risk factor as previously reported.^21^ The authors of the present study hypothesize that this is due to the perception that these young mothers will require more help, and the nursing staff and family support system after discharge is sensitized to help after the initial discharge.

The findings of this study have to be seen in the light of some limitations. This is a,single-center, retrospective study, and therefore its conclusions could be affected by local practices. Also, there may be a small number of infants readmitted to nearby county hospitals more adjacent to the parents’ place of residence; however, empirically, the authors found that parents are more inclined to present newborn infants to the hospital of birth in thec region the authors focused on.

In conclusion, readmission rates in the neonatal period for infants born at ≥36 weeks gestation and admitted to a well-baby nursery in the studied cohort is 4.0% and is similar to those reported earlier. In contrast to other studies, respiratory tract infections are the leading cause of readmission, followed by jaundice. The present study's data suggest that length of initial hospitalization should not be the main focus of discussion in the prevention of readmission in the neonatal period, but more emphasis needs to be placed on providing expert staff support to the family of the infant. In this light, the authors’ finding that infants transferred to special care or a NICU setting during the initial hospitalization were less likely to require hospitalization in the neonatal period is an interesting one. Further research into how different approaches in these two settings reduce the risk of readmission is needed.

## Author's contributions

Kardum D and Serdarušić I made substantial contributions to the conception and design of the study, data acquisition, analysis and interpretation, manuscript drafting and critical review for important intellectual content. Biljan B, Šantić K, Živković V made substantial contributions to the data acquisition, analysis and interpretation, manuscript drafting and critical review for important intellectual content. All of the authors have approved the final version of the manuscript.

## Conflicts of interest

The authors declare no conflicts of interest.

## References

[1] Oddie SJ, Hammal D, Richmond S, Parker L. (2005). Early discharge and readmission to hospital in the first month of life in the Northern Region of the UK during 1998: a case cohort study. Arch Dis Child.

[2] Meara E, Kotagal UR, Atherton HD, Lieu TA. (2004). Impact of early newborn discharge legislation and early follow-up visits on infant outcomes in a state Medicaid population. Pediatrics.

[3] Jones E, Stewart F, Taylor B, Davis PG, Brown SJ (2021). Early postnatal discharge from hospital for healthy mothers and term infants. Cochrane Database Syst Rev.

[4] Farhat R, Rajab M. (2011). Length of postnatal hospital stay in healthy newborns and re-hospitalization following early discharge. N Am J Med Sci.

[5] Habib HS. (2013). Impact of discharge timings of healthy newborns on the rates and etiology of neonatal hospital readmissions. J Coll Physicians Surg Pak.

[6] Perme T, A Škafar Cerkvenik, Š Grosek (2016). Newborn readmissions to slovenian children's hospitals in one summer month and one autumn month: a retrospective study. Pediatr Neonatol.

[7] Escobar GJ, Greene JD, Hulac P, Kincannon E, Bischoff K, Gardner MN (2005). Rehospitalisation after birth hospitalisation: patterns among infants of all gestations. Arch Dis Child.

[8] Braveman P, Egerter S, Pearl M, Marchi K, Miller C. (1995). Problems associated with early discharge of newborn infants. Early discharge of newborns and mothers: a critical review of the literature. Pediatrics.

[9] Harrold J, Langevin M, Barrowman N, Sprague AE, Fell DB, Moreau KA (2018). Pediatric Emergency Research Canada Network. Parental characteristics and perspectives pertaining to neonatal visits to the emergency department: a multicentre survey. CMAJ Open.

[10] Thilo EH, Townsend SF, Merenstein GB. (1998). The history of policy and practice related to the perinatal hospital stay. Clin Perinatol.

[11] Campbell OM, Cegolon L, Macleod D, Benova L. (2016). Length of stay after childbirth in 92 countries and associated factors in 30 low- and middle-income countries: compilation of reported data and a cross-sectional analysis from nationally representative surveys. PLoS Med.

[12] Luciano R. (2015). Early discharge of term neonates: we can do it safely. Ital J Pediatr.

[13] Datar A, Sood N. (2006). Impact of postpartum hospital-stay legislation on newborn length of stay, readmission, and mortality in California. Pediatrics.

[14] Paul IM, Lehman EB, Hollenbeak CS, Maisels MJ. (2006). Preventable newborn readmissions since passage of the Newborns' and Mothers' Health Protection Act. Pediatrics.

[15] Metcalfe A, Mathai M, Liu S, Leon JA, Joseph KS. (2016). Proportion of neonatal readmission attributed to length of stay for childbirth: a population-based cohort study. BMJ Open.

[16] Kavehmanesh Z, Mohammadieh NE, Zarchi AK, Amirsalari S, Matinzadeh ZK, Torkaman M. (2008). Prevalence of readmission for hyperbilirubinemia in healthy newborns. Iran J Pediatr.

[17] Young PC, Korgenski K, Buchi KF. (2013). Early readmission of newborns in a large health care system. Pediatrics.

[18] Hall RT, Simon S, Smith MT. (2000). Readmission of breastfed infants in the first 2 weeks of life. J Perinatol.

[19] American Academy of Pediatrics (2010). Committee on fetus and newborn. Hospital stay for healthy term newborns. Pediatrics.

[20] Blumovich A, Mangel L, Yochpaz S, Mandel D, Marom R. (2020). Risk factors for readmission for phototherapy due to jaundice in healthy newborns: a retrospective, observational study. BMC Pediatr.

[21] Martens PJ, Derksen S, Gupta S. (2004). Predictors of hospital readmission of Manitoba newborns within six weeks postbirth discharge: a population-based study. Pediatrics.

